# Creating a Single Inflow Orifice From Living Donor Kidney Allografts With Multiple Renal Arteries

**DOI:** 10.3389/ti.2022.10212

**Published:** 2022-04-14

**Authors:** Marina M. Tabbara, Giselle Guerra, Juliano Riella, Phillipe Abreu, Angel Alvarez, Rodrigo Vianna, Linda Chen, Mahmoud Morsi, Jeffrey J. Gaynor, Javier Gonzalez, Gaetano Ciancio

**Affiliations:** ^1^ Department of Surgery, Miller School of Medicine, University of Miami, Miami, Florida; ^2^ Miami Transplant Institute, Miller School of Medicine, University of Miami, Jackson Memorial Hospital, Miami, Florida; ^3^ Division of Nephrology, Department of Medicine, Miller School of Medicine, University of Miami, Miami, Florida; ^4^ Department of Urology, Hospital General Universitario Gregorio Marañón, Madrid, Spain; ^5^ Department of Urology, Miller School of Medicine, University of Miami, Miami, Florida

**Keywords:** multiple renal blood vessels, surgical innovation, living donors, renal transplantation, kidney allografts

## Abstract

**Background:** Multiple renal arteries (MRA) are often encountered during living-donor kidney transplantation (LDKT), requiring surgeons to pursue complex renovascular reconstructions prior to graft implantation. With improvements in reconstruction and anastomosis techniques, allografts with MRA can be successfully transplanted with similar outcomes to allografts with a single renal artery. Here, we describe in detail various surgical techniques for reconstruction of MRA grafts with the intent of creating a single arterial inflow.

**Methods:** We retrospectively reviewed the medical records of all LDKT recipients with laparoscopically procured MRA kidneys between March 2008 and July 2021. Recipient and donor characteristics, operative data, type of reconstruction, and recipient outcomes were analyzed. The primary outcomes were the incidence of developing delayed graft function (DGF) and/or a vascular or urological complication within 12 months post-transplant.

**Results:** Seventy-three LDKT recipients of MRA donor allografts were evaluated. Two renal arteries (RA) were encountered in 62 allografts (84.9%) and three RA in 11 allografts (15.1%). Renal artery reconstruction was performed in 95.8% (70/73) of patients. Eighteen different reconstruction techniques of MRA were utilized, the most common being side-to-side anastomosis in allografts with two RA (*N* = 44) and side-to-side-to-side anastomosis in allografts with three RA (*N* = 4). Interposition grafting was performed in seven cases (9.6%). A single ostium was created in 69 cases (94.5%), and the median warm ischemia time was 27 (range 20–42) minutes. None of the patients developed DGF or post-operative vascular or urological complications. Median creatinine at 3, 6, and 12 months post-transplant remained stable at 1.1 mg/dl. With a median follow-up of 30.4 months post-transplant, only one graft failure has been observed–death-censored graft survival was 98.6%.

**Conclusion:** Complex reconstruction techniques to create a single renal artery ostium for graft implantation anastomosis in allografts with MRA show acceptable warm ischemic times, with no increased risk of post-operative vascular or urological complications.

## Graphical Abstract

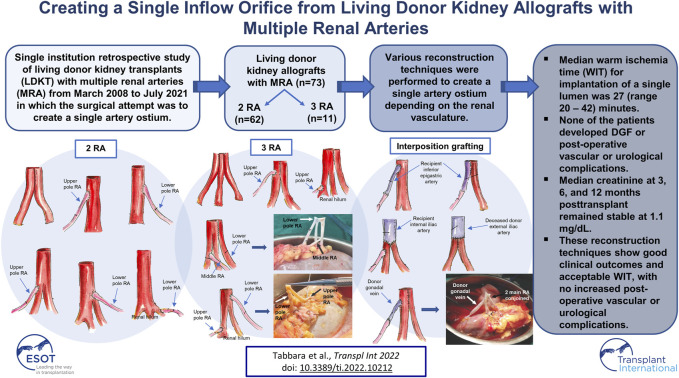

## Introduction

With a widening gap between supply and demand of organs, living-donor kidney transplantation (LDKT) has substantially increased in efforts to expand the donor pool. This has led to a surge in living-donor kidneys (LDK) with anatomical variations, specifically, multiple renal arteries (MRA) (1, 2). Kidneys with MRA are common in renal vascular anatomy, occurring at an incidence of 18–43% in potential kidney donors (3). When encountered during LDKT, they often require complex back-table reconstructions, which has been associated with a higher risk of post-transplant vascular and urologic complications (1, 4, 5). However, with improvements in reconstruction and anastomosis techniques, allografts with MRA have been shown to be successfully transplanted with similar surgical and clinical outcomes compared to allografts with a single renal artery (6–8). Examples of these improvements include the use of interposition grafting (9, 10) and side-to-side anastomoses to create a wide lumen (11, 12). Additionally, routine use of low-molecular weight dextran and optical magnification have helped to minimize postoperative complications and made it easier to construct microvascular anastomosis during LDKT (6–8).

Although long-term graft and patient survival have been shown to be similar for single and multiple arteries, the impact of the type of arterial reconstruction method for MRA has rarely been investigated and warrants additional study (12, 13). Performing multiple anastomoses is often associated with poor visibility, difficult suturing (14), thrombosis, and bleeding (15). Additionally, multiple anastomoses are associated with a prolonged warm ischemia time (WIT), which has been shown to have a detrimental effect on both early graft function and long-term graft survival in LDKT (16–21). In this study, we describe in detail 18 different surgical techniques for reconstruction of MRA during LDKT, with the main goal of creating a single renal artery ostium for allograft implantation in efforts to facilitate construction of the *in situ* vascular anastomosis, minimize recipient WIT, and reduce post-operative complications. We evaluated recipient and donor demographics, operative data, early outcomes such as delayed graft function (DGF), development of any post-operative vascular, urological, or other complication within 12 months post-transplant, and graft survival.

## Methods

We retrospectively reviewed the medical records of all LDKT recipients with laparoscopically procured MRA kidneys at our institution between March 2008 and July 2021. This study was approved by the University of Miami Institutional Review Board and follows the ethical principles (as revised in 2013) of the Helsinki Declaration. All patients gave written informed consent prior to enrollment.

All donors underwent comprehensive nephrologic evaluation including their medical history, physical examination, renal function assessment, and urinalysis. Evaluation of the donor renal vascular anatomy was performed using computed tomography angiography (CTA). Thus, the presence of multiple vessels was known before surgery. All donors referred to us were considered suitable based on their vasculature. The approach for reconstruction of MRA and the availability of deceased donor vessels for interposition grafting reconstruction were determined before surgery.

All recipients began Aspirin 81 mg daily on post-operative day 3 and remained on this regimen indefinitely. To monitor development of vascular and/or urological complications, baseline Doppler Ultrasound (DU) was performed after surgery, and then repeated at 1, 3, and 12 months post-operatively. If there were any vascular or urological concerns, further imaging with magnetic resonance angiography and/or Tc99m MAG-3 renal scintigraphy was performed.

### Statistical Analysis

Analyzed baseline variables included date of transplant, recipient age, recipient gender, recipient race/ethnicity, recipient BMI, recipient pre-transplant history of diabetes mellitus, kidney retransplant status, donor type, donor kidney location (left or right), number of donor arteries, type of vascular reconstruction, whether or not a single renal artery ostium was used, living donor type (related/unrelated), double-J ureteral stent insertion, JP drain insertion, total operative time, cold ischemia time (CIT), and warm ischemia time (WIT) for single and multiple anastomoses. Recipient outcomes included development of DGF (requirement for dialysis during the first post-operative week), length of hospital stay, development of a post-operative vascular, urological, or other complication within 12 months posttransplant, and graft loss (return to permanent dialysis or death). Estimated glomerular filtration rate (eGFR) was calculated using the Chronic Kidney disease Epidemiology Collaboration Equation. Percentages of patients having selected baseline characteristics were determined as well as means, standard errors, medians, and ranges of values for baseline continuous variables.

### Surgical Techniques

A hand-assisted laparoscopic donor nephrectomy was performed in a standard fashion with special attention given to the renal hilum and preservation of the length of the renal vessels (22). The vessels were stapled using the Ethicon Echelon Flex Powered Stapler with the 45-mm vascular linear cutter. In the case of early bifurcation, we used the Ethicon Echelon Flex Powered Stapler with a 35-mm vascular linear cutter to avoid having two renal vessels. The graft was flushed with cold Histidine-tryptophan-ketoglutarate until the effluent was clear. The renal arteries and veins were dissected from the surrounding perivascular lymphatics and fat. The donor and recipient vessels were prepared by trimming any redundant length of the vessels to prevent kinking during anastomosis. The ureter with its blood supply and the periureteric tissue were preserved, and all remaining redundant perinephric fat was trimmed.

*Ex-vivo* reconstructions were performed during bench surgery according to the case-specific anatomy. Surgical loupes at 3.5x magnification were used for the reconstructions. All the vascular reconstructions were performed with 8–0 Prolene.

**FIGURE 1:**
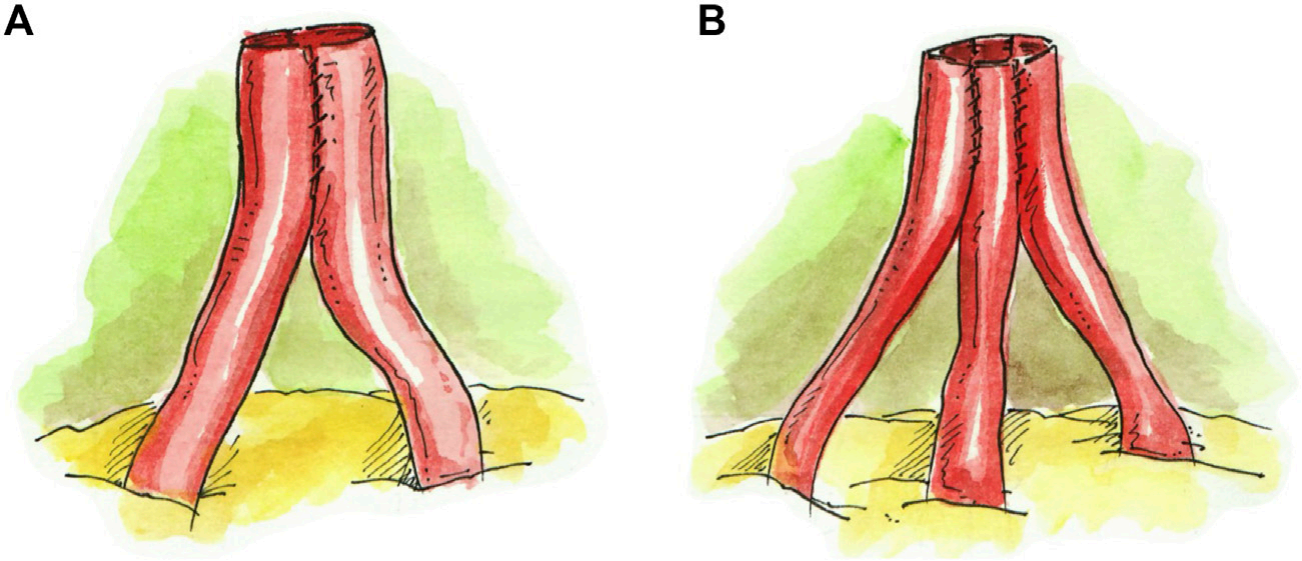
Conjoined anastomosis techniques. **(A)** Single ostium side-to-side anastomosis. **(B)** Single ostium side-to-side-to-side anastomosis.

In the case of two renal arteries (RA) of similar length (*N* = 43), the preferred approach was a single ostium side-to-side anastomosis, which was created by spatulating the two arteries medially and conjoining them into a single lumen (Figure 1A). This technique was extrapolated in the case of three RA of similar length (*N* = 4), where a single renal artery ostium was created by conjoining the arteries in a side-to-side-to-side manner (Figure 1B). If the additional renal artery was <1 mm in length and not suitable for anastomosis, it was tied off, and the remaining two RA were conjoined together into a single lumen (N = 1) (Figure 1A).

**FIGURE 2:**
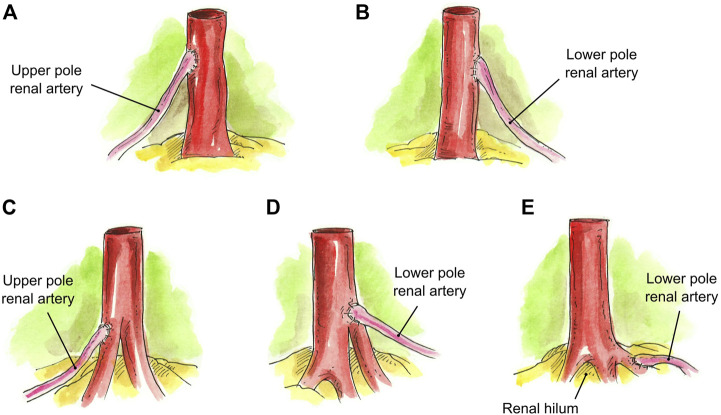
Techniques for grafting a main RA and an accessory pole artery. **(A)** UPRA anastomosed end-to-side to main RA. **(B)** LPRA anastomosed end-to-side to main RA. **(C)** Short UPRA anastomosed end-to-side to a branch of the main RA. **(D)** Short LPRA anastomosed end-to-side to a branch of the main RA. **(E)** Short LPRA anastomosed end-to-side to a branch of the main RA inside the hilum.

In the case of a graft with a main RA and an accessory upper pole renal artery (UPRA) (*N* = 4) or lower pole renal artery (LPRA) (*N* = 3), an end-to-side anastomosis to the main RA was created in a running fashion (Figures 2A,B). If there was a short UPRA (*N* = 1) or short LPRA (*N* = 1), it was anastomosed end-to-side to one of the branches of the main RA (Figures 2C,D). In one case, the short LPRA was anastomosed to a branch of the main RA inside the hilum (*N* = 1) (Figure 2E).

**FIGURE 3:**
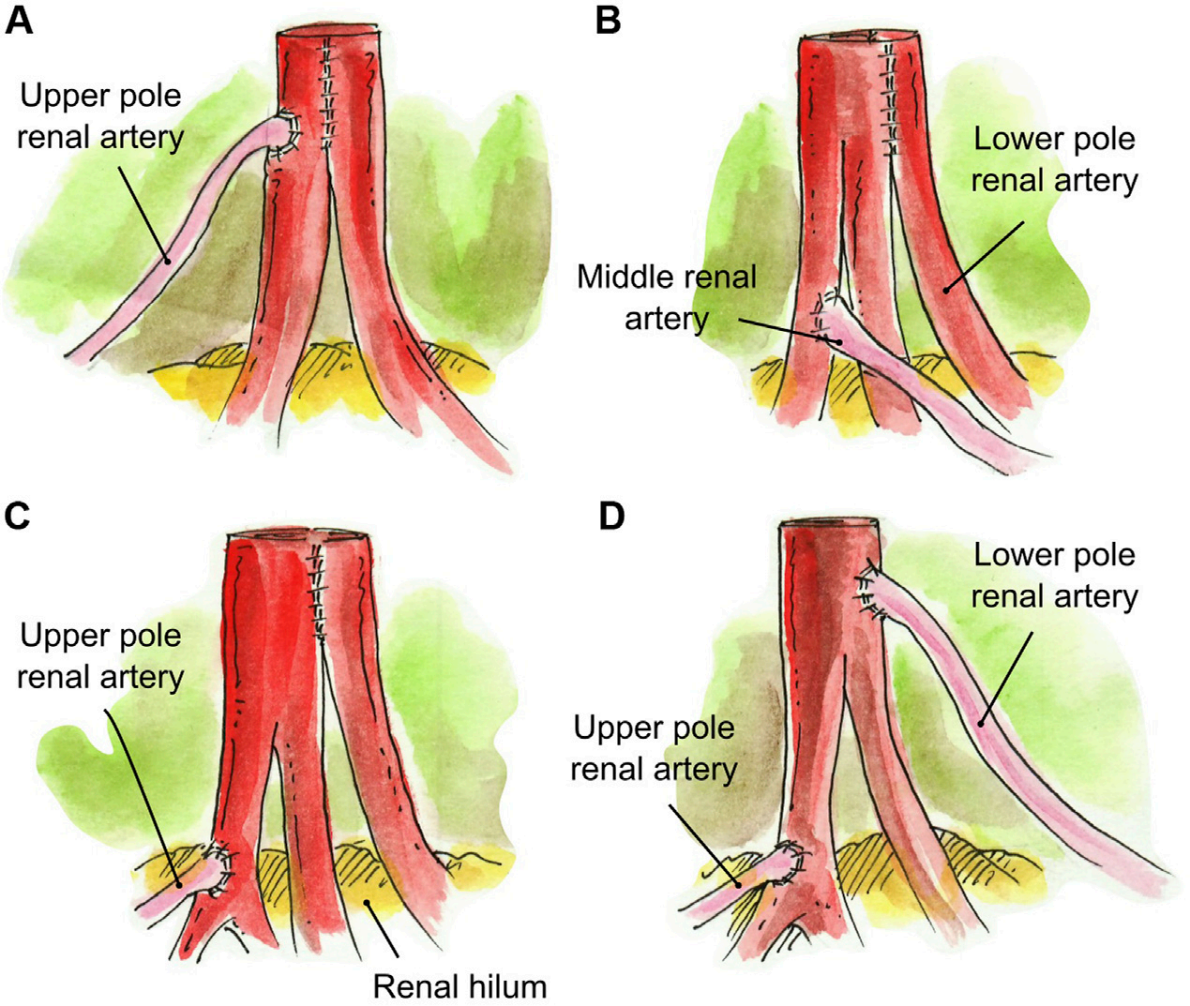
Creation of a single inflow orifice for grafts with 3RA. **(A)** Two main RA conjoined side-to-side and UPRA anastomosed end-to-side to the upper main RA. **(B)** LPRA conjoined in a single lumen with the main RA, and short middle RA anastomosed end-to-side to the upper branch of the main RA. **(C)** Two RA conjoined side-to-side in a single lumen and short UPRA anastomosed end-to-side to a branch of the upper renal artery inside the hilum. **(D)** Short UPRA anastomosed end-to-side to a branch of the main RA inside the hilum, and the LPRA was anastomosed end-to-side to main RA.

In the case of three RA, several approaches were taken to create a single ostium. One approach was to conjoin the two main RA side-to-side and then anastomose the UPRA end-to-side to the upper main RA (*N* = 1) (Figure 3A). In one case, the LPRA was conjoined in a single lumen with the main RA, and the middle RA was anastomosed end-to-side to the upper branch of the main RA (*N* = 1) (Figures 3B, 6A). In another approach, the two main RA were anastomosed side-to-side in a single lumen and the short UPRA was anastomosed end-to-side to a branch of the RA inside the hilum (*N* = 1) (Figure 3C). Finally, there was one case where the short UPRA was anastomosed end-to-side to a branch of the main RA inside the hilum, and the LPRA was anastomosed end-to-side to main RA (*N* = 1) (Figures 3D, 6B).

**FIGURE 4:**
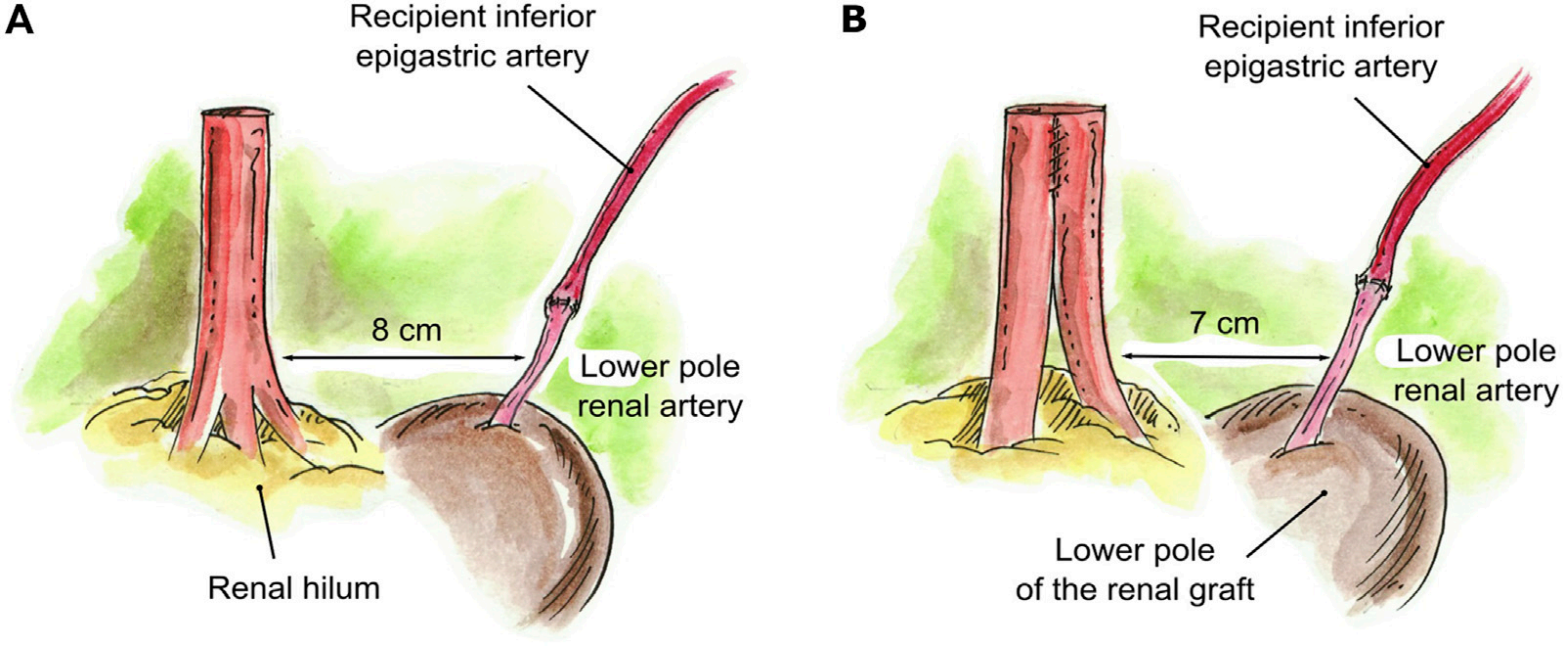
Creation of two separate anastomoses for implantation. **(A)** Short LPRA (8 cm from the main RA) anastomosed end-to-end to the recipient inferior epigastric artery. **(B)** Two RA were conjoined in a single lumen and the LPRA (7 cm from the 2 RA) anastomosed end-to-end to the recipient ipsilateral inferior epigastric artery.

When an accessory pole artery was located too far from the renal artery(s) and creation of a single ostium was not feasible, two separate arterial anastomoses were implanted (*N* = 4). In three of these cases, there were grafts with two RA, with a short LPRA located too far from the main RA to perform a reconstruction. One of these was a case of 2-year-old pediatric recipient in which the LPRA was anastomosed end-to-side to the external iliac artery, and the main RA was anastomosed end-to-side to the common iliac artery. The two remaining cases had a short LPRA that was 8 cm from the main RA. The short LPRA was anastomosed end-to-end to the recipient inferior epigastric artery, and the main RA was anastomosed end-to-side to the external iliac artery (Figure 4A). In the final case of a graft with three RA, a LPRA was 7 cm from the two main RA. The two main RA were conjoined together side-to-side into a single ostium, and the short LPRA was anastomosed end-to-end to the recipient ipsilateral inferior epigastric artery, which was fully mobilized and dissected from the abdomen (Figure 4B).

**FIGURE 5:**
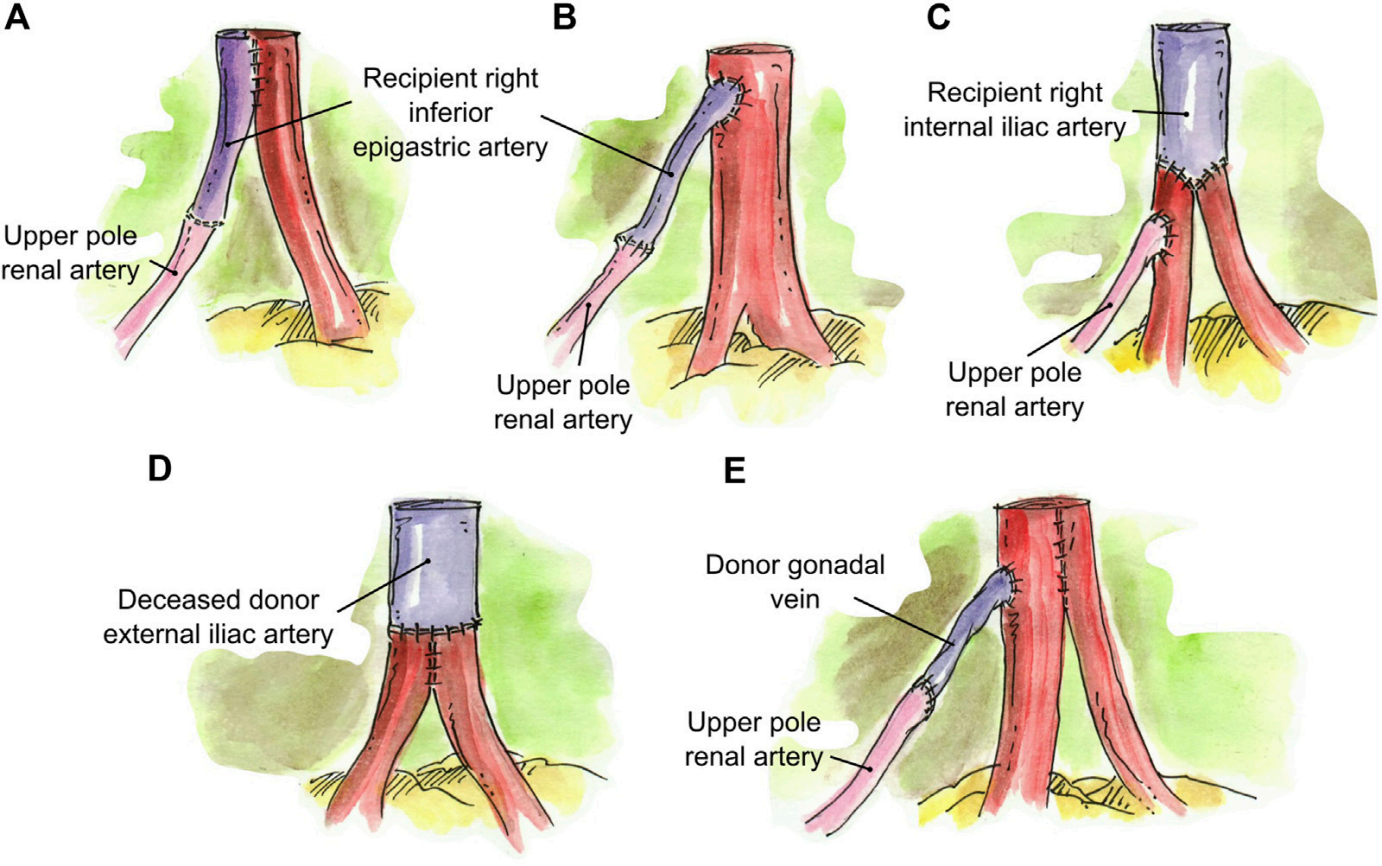
Interposition grafting. **(A)** Segment of recipient inferior epigastric artery anastomosed end-to-end to the short UPRA, and then anastomosed side-to-side to the main RA. **(B)** Segment of recipient inferior epigastric artery anastomosed end-to-end to the short UPRA, and then anastomosed end-to-side to the main RA. **(C)** Segment of recipient internal iliac artery anastomosed end-to-end to the two main RA, and the short UPRA anastomosed end-to-side to one of the main RA. **(D)** Segment of deceased donor external iliac artery anastomosed end-to-end to two short RA conjoined in a single lumen. **(E)** Short UPRA extended with a segment of donor gonadal vein, then anastomosed end-to-side to the one of the 2 main RA that were conjoined in single ostium.

**FIGURE 6:**
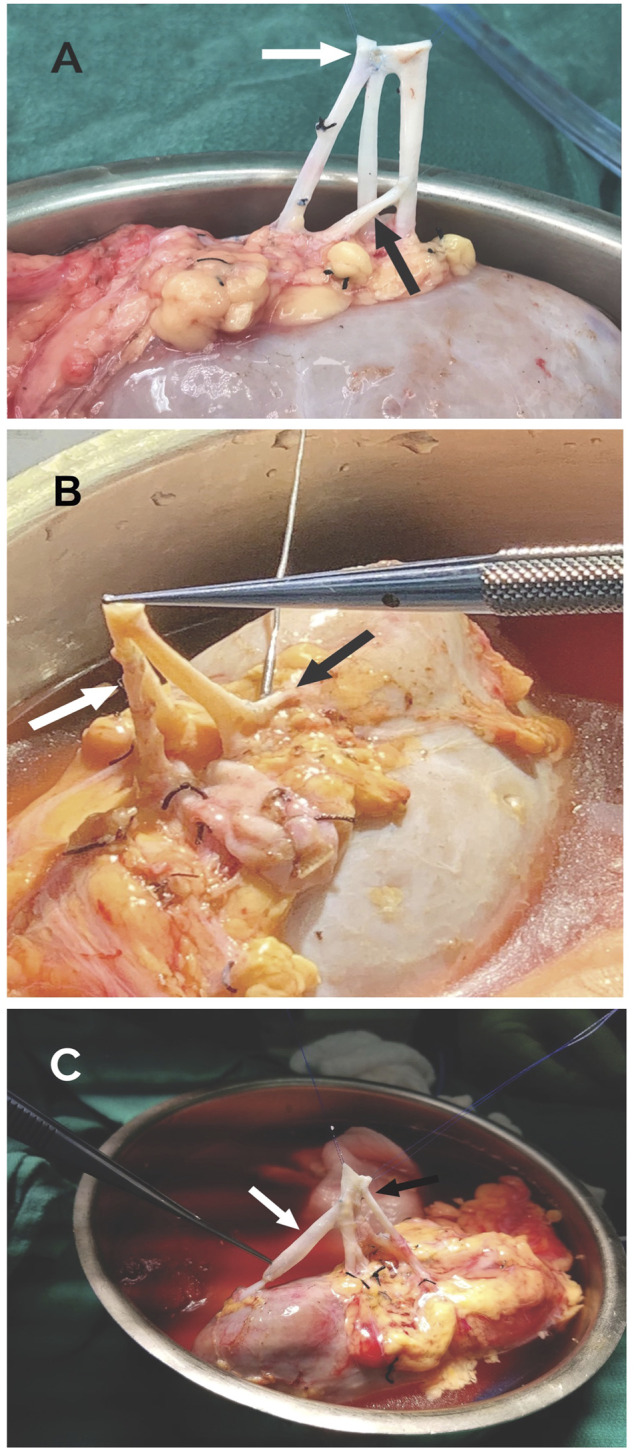
**(A)** LPRA (white arrow) anastomosed side-to-side the main RA with 8–0 Prolene, middle RA anastomosed end-to-side to the main RA (black arrow) with 8–0 Prolene. **(B)** LPRA (white arrow) anastomosed end-to-side to the main RA with 8–0 Prolene. The UPRA was short, so it was anastomosed end-to-side to one of the branches of the main RA inside the hilum. **(C)** UPRA anastomosed end-to-end to the donor gonadal vein with 8–0 Prolene, then end-to-side with 8–0 Prolene to the main RA (white arrow). The 2 RA were conjoined side-to side with 8–0 Prolene (black arrow).

Interposition grafting was utilized as various conduits for short renal arteries. A segment of recipient inferior epigastric artery (RIEA) was used in four renal grafts two 2 RA; a short UPRA was anastomosed end-to-end to the RIEA, and then anastomosed side-to-side (*N* = 3) or end-to-side (*N* = 1) to the main RA (Figures 5A,B). In a graft with three RA, the two main RA were anastomosed end-to-end to a segment of the recipient internal iliac artery, and the short UPRA was anastomosed end-to-side to one of the main RA (*N* = 1) (Figure 5C). A segment of deceased donor external iliac artery was used to extend two short RA conjoined in a single lumen (N = 1) (Figure 5D). Finally, a segment of donor gonadal vein was used to extend a short UPRA in a graft with three RA, which was anastomosed end-to-side to the one of the two main RA that were conjoined into single ostium (*N* = 1) (Figures 5E, 6C).

Once the reconstructions were complete, grafts were anastomosed end-to-side to the recipient external iliac artery and vein. After reperfusion, an extravesical ureteroneocystostomy was performed (23).

Of note, while diameter sizes of donor arteries were measured pre-operatively by CTA (upper and lower pole arteries measured approximately 2 mm in diameter), the diameter of the ostium of the reconstructed arteries was not measured. However, its diameter was the combined size of the two or three conjoined RA.

### Immunosuppression

All recipients received immunosuppressant therapy according to our center’s protocols (24) with induction consisting of intravenous antithymocyte globulin 1 mg/kg, basiliximab 20 mg, and methylprednisolone 500 mg administered intraoperatively before organ reperfusion. Maintenance immunosuppression included a steroid-free regimen consisting of tacrolimus and mycophenolate mofetil, starting on postoperative day 1.

## Results

Recipient and donor baseline demographics and operative data appear in Table 1. Seventy-three LDKT recipients of MRA donor allografts were evaluated. Median recipient age was 48.8 (range 2.3–77.1) years, and 67.1% (49/73) recipients were male. Black and Hispanic participants comprised 12.3% (9/73) and 42.5% (31/73) of the transplant recipients, respectively. The majority of transplant recipients, 93.2% (68/73), received a primary kidney transplant; only 6.8% (5/73) were retransplants. The percentage of recipients who received a left donor kidney was 94.5% (69/73); 5.5% (4/73) received a right donor kidney. The percentage who received a kidney with two RA and three RA was 83.6% (61/73) and 16.4% (12/73), respectively. A double-J ureteral stent was placed in only 4.1% (3/73) of the patients. A JP drain was placed in 20.5% (15/73) of the patients. Median total operative time was 296 (range 206–483) minutes. The median warm ischemia time for anastomosis of a single artery ostium was 27 (range 20–42) minutes, and for two separate anastomoses it was 31.5 (range 21–33) minutes. Median estimated blood loss was 40 (range 10–300) ml.

**TABLE 1:** Distributions of recipient and donor demograpghics and of recipient operative data.

Baseline variable	Mean ± SE if continuous (geometric mean ± SE for variables with skewed distributions); Percentage with characteristic if categorical
Recipient age (year)	47.2 ± 1.9 (N = 73)
—	(Median = 48.8, Range: 2.3–77.1)
Recipient age (year)	—
<18	6.8% (5/73)
≥18, <50	43.8% (32/73)
≥50	49.3% (36/73)
Recipient Gender	—
Female	32.9% (24/73)
Male	67.1% (49/73)
Recipient race/Ethnicity	—
Black (non-Hispanic)	12.3% (9/73)
Hispanic	42.5% (31/73)
White (non-Hispanic)	41.1% (30/73)
Other	4.1% (3/73)
Recipient BMI (kg/m^2^)	26.3 ± 0.7 (*N* = 73)
	(Median = 26.0, Range: 16.0–42.4)
Recipient pretransplant diabetes mellitus	—
No	76.7% (56/73)
Yes	23.2% (17/73)
Retransplant	—
No	93.2% (68/73)
Yes	6.8% (5/73)
Donor type	—
Living related	57.5% (42/73)
Living unrelated	42.5% (31/73)
Kidney	—
Left	94.5% (69/73)
Right	5.5% (4/73)
Number of Donor arteries	—
2	83.6% (61/73)
3	16.4% (12/73)
JP drain placed	—
No	79.5% (58/73)
Yes	20.5% (15/73)
Double-J ureteral stent placed	—
No	95.9% (70/73)
Yes	4.1% (3/73)
Total Operative Time (min)	309.2 ± 8.1 (*N* = 73)
—	(Median = 296, Range: 206–483)
CIT (min)	77.8 ± 2.9 (*N* = 73)
—	(Median = 73, Range: 15–190)
WIT (min)	28.2 ± 0.6 (*N* = 73)
—	(Median = 27, Range: 20–42)
WIT single anastomosis (min)	28.1 ± 0.6 (*N* = 69)
—	(Median = 27, Range: 20–42)
WIT two anastomosis (min)	29.3 ± 2.8 (*N* = 4)
—	(Median = 31.5, Range: 21–33)
EBL (ml)	37.9 */1.09 (*N* = 73)
—	(Median = 40.0, Range: 10–300)

The types of reconstruction are detailed in Table 2. Renal artery reconstruction was performed in 95.8% (70/73) of patients; reconstruction was not performed in three patients. A single renal artery ostium was created in 94.5% (69/73) of patients. Two separate renal artery anastomoses were implanted in 5.5% (4/73) of patients. Interposition grafting was performed in seven cases (9.6%).

**TABLE 2:** Types of reconstruction.

2 RA (*N* = 61)	N (%)
None^a^ (Figure 4A and one pediatric case not illustrated)	3 (4.9%)
Conjoined, side-to-side (Figure 1A)	43 (70.4%)
Accessory pole RA end-to-side to main RA (Figures 2A, 2B)	7 (11.5%)
Accessory pole RA end-to-side to branch of main RA (Figures 2C, 2D)	2 (3.3%)
Accessory RA end-to-side to branch of main RA inside the hilum (Figure 2E)	1 (1.5%)
UPRA end-to-end to Recipient IEA,^b^then either side-to-side or end-to-side to main RA (Figures 5A, 5B)	4 (6.6%)
2 conjoined RA end-to-end to a segment of Deceased Donor EIA^b^ (Figure 5D)	1 (1.5%)
**3 RA (*N* = 12)**	
Accessory pole <1 mm ligated, 2 remaining RA conjoined side-to-side (Figure 1A)	1 (1.5%)
Conjoined, side-to-side-to-side (Figure 1B)	4 (36.4%)
2 RA conjoined, UPRA end-to-side to main RA (Figure 3A)	1 (8.3%)
LPRA and main RA conjoined, middle RA end-to-side to upper branch of main RA (Figures 3B, 6A)	1 (8.3%)
2 RA conjoined side-to-side, UPRA end-to-side to branch of upper RA inside the hilum (Figure 3C)	1 (8.3%)
LPRA end-to-side to main RA, UPRA end-to-side to branch of RA inside the hilum (Figures 3D, 6B)	1 (8.3%)
2 RA conjoined, and LPRA end-to-end to recipient IEA^a^ (Figure 4B)	1 (8.3%)
2 main RA end-to-end to a segment of Recipient IIA, then UPRA end-to-side to one of the main RA (Figure 5C)	1 (8.3%)
UPRA end-to-end to a segment of Donor gonadal vein,^b^ then end-to-side to 2 conjoined RA (Figures 5E, 6C)	1 (8.3%)

a Two separate anastomosis.

b Interposition grafting.

Abbreviations: IEA = inferior epigastric artery; EIA = external iliac artery; UPRA= upper pole renal artery; LPRA = lower pole renal artery; IIA = internal iliac artery.

Recipient outcomes are listed in Table 3. Median length of hospital stay was 4 (range 3–67) days. Median follow-up among 67 patients who were alive with a functioning graft as of the last follow-up date (31 July 2021) was 30.4 (range: 0.3–151.2) months post-transplant. Median preoperative creatinine was 6.0 (range 0.9–22.6) mg/dl, which decreased to 1.1 (range 0.25–2.0) mg/dl at 3 months. At 6 and 12 months post-transplant, the median creatinine remained stable at 1.1 mg/dl.

**TABLE 3:** Recipient outcomes.

Outcome variable	Mean ± SE if continuous (geometric mean ± SE for variables with skewed distributions); Percentage with characteristic if categorical
Length of hospital stay (days)	4.71 ± 1.06 (*N* = 73)
—	(Median = 4, Range: 3–67)
Developed delayed graft function (DGF)	—
No	100.0% (73/73)
Yes	0.0% (0/73)
Developed a post-operative complication (vascular, urological, or surgical) (within 12 months post-transplant)^a^	—
No	97.3% (71/73)
Yes	2.7% (2/73)
	—
Serum Cr at DOT (mg/dl)	6.9 ± 1.07 (*N* = 73)
—	(Median = 6.0, Range: 0.9–22.6)
Serum Cr at 3 months post-tx (mg/dl)	1.1 ± 1.04 (*N* = 67)
—	(Median = 1.1, Range: 0.25–2.0)
Serum Cr at 6 months post-tx (mg/dl)	1.1 ± 1.04 (*N* = 65)
—	(Median = 1.1, Range: 0.3–2.0)
Serum Cr at 12 months post-tx (mg/dl)	1.2 ± 1.05 (N = 60)
—	(Median = 1.1, Range: 0.3–4.9)
eGFR at 3 months post-tx (ml/min/1.73 m^2^)	78.4 ± 3.4 (*N* = 67)
—	(Median = 76.8, Range: 34.8–234.5)
eGFR at 6 months post-tx (ml/min/1.73 m^2^)	76.5 ± 3.3 (*N* = 65)
—	(Median = 74.2, Range: 38.2–217.2)
eGFR at 12 months post-tx (ml/min/1.73 m^2^)	76.2 ± 3.7 (*N* = 60)
—	(Median = 70.9, Range: 15.6–216.5)
eGFR at 36 months post-tx (ml/min/1.73 m^2^)	66.8 ± 4.2 (*N* = 30)
—	(Median = 66.6, Range: 12.0–114.0)
eGFR at 60 months post-tx (ml/min/1.73 m^2^)	62.6 ± 6.2 (*N* = 18)
—	(Median = 67.5, Range: 6.2–107.7)
Graft failure, (i.e., return to permanent dialysis or retransplanted) (as of the Last follow-up date)^b^	—
No	98.6% (72/73)
Yes	1.4% (1/73)
Death with a functioning graft (as of the last follow-up date)^b^	—
No	93.2% (68/73)
Yes	6.8% (5/73)
Graft Loss (death uncensored) (as of the last follow-up date)^b^	—
No	91.8% (67/73)
Yes	8.2% (6/73)

a Among the 2 patients who developed a post-operative complication during the first 12 months post-transplant, the following complications were observed: acute respiratory distress syndrome (ARDS) (N = 1), and *C. difficile* colitis/sepsis (*N* = 1).

b The date of last follow-up for this study was 31 July 2021. Median follow-up among 67 patients who were alive with a functioning graft as of the last follow-up date was 30.4 (range: 0.3–151.2) months post-transplant. The single cause and time-to-graft failure (return to permanent dialysis) was as follows (listed chronologically by time to graft failure): Acute TCMR, at 41.8 months post-transplant. The 5 causes of death with a functioning graft and times-to-death were as follows: Cardiovascular Event in 2 patients (at 4.4- and 7.9-months post-transplant, respectively), Infection in 2 patients (death due to *C. difficile* colitis/sepsis in 1 patient at 0.8 months post-transplant, and death due to infection/sepsis in 1 patient at 125.2 months post-transplant), and Ruptured Aortic Aneurysm in 1 patient (at 5.2 months post-transplant).

None of the 73 patients had DGF or developed a postoperative vascular or urological complication. Since the main concern with lower pole artery reconstruction is the risk of developing a postoperative urological complication, it was reassuring that no such complication was observed in any of the patients. Thus, there were no differences in clinical outcomes between those who received an upper pole artery vs. lower pole artery reconstruction.

Two patients (2.7%) developed a nonsurgical post-operative complication during the first 30 days (12 months) post-transplant, including *C. difficile* colitis/sepsis at 4 days post-transplant (*N* = 1) and acute respiratory distress syndrome (ARDS) at 6 days post-transplant (*N* = 1). The patient who developed *c. difficile* colitis/sepsis died of that infection (with a functioning graft) at 0.8 months post-transplant. The patient who developed ARDS did not experience graft loss.

One patient (1.4%) developed graft failure due to acute T-cell-mediated rejection (TCMR) at 41.8 months post-transplant. Five patients have died with a functioning graft: cardiovascular event in two patients (at 4.4 and 7.9 months post-transplant, respectively), infection in two patients (death due to *C. difficile* colitis/sepsis in one patient at 0.8 months post-transplant, and death due to sepsis in one patient at 125.2 months post-transplant), and ruptured aortic aneurysm in one patient (at 5.2 months post-transplant).

## Discussion

Kidney transplantation is the treatment of choice for patients with ESRD. However, donor organ shortage has prevented the wider application of this treatment. This has prompted surgeons to utilize each donor organ they encounter in a maximal and favorable manner, such as kidney grafts with MRA (7, 25). Up until recently, renal artery multiplicity was viewed as a contraindication for transplantation due to its greater technical demand and association with a higher incidence of vascular and urological complications (1, 4, 5). Additionally, prolonged total operative times and ischemia times were thought to add unnecessary risk to the recipient (7, 26). However, with recent advances in surgical reconstruction and anastomoses techniques, transplantation of allografts with MRA is no longer considered to be a surgical restriction and has been shown to provide comparable post-operative and clinical outcomes to allografts with a single renal artery (5, 7, 8, 27, 28).

Several reconstruction techniques of MRA have been described in the literature with the common goal of minimizing ischemic insult and avoiding vascular complications. Transplantation of MRA in LDKT is often achieved by performing multiple arterial anastomoses without reconstruction. In a retrospective study by Hwang et al, sequential arterial anastomoses of MRA were performed in 81.1% of their cases with MRA; the remaining grafts with MRA were implanted with single anastomosis by either conjoining the renal arteries into a single lumen or ligating the accessory polar artery (29). Vaccarisi et al explained that in cases of MRA, they did not consider the opportunity to perform vascular reconstruction to unify the ostium, and all anastomoses were created separately in succession without kidney reperfusion (30). Popov et al mentioned that when dealing with two arteries of unequal size, it is preferable to anastomose them separately rather than to perform bench surgery, thereby decreasing the risk of compromising the lumen of the larger renal artery (31).

Although multiple anastomoses techniques like those described can provide good long-term outcomes, they are often associated with poor visibility and difficult suturing (14). We believe it is advantageous to create a single arterial lumen from MRA while in cold preservation, as it facilitates *in situ* vascular anastomosis and minimizes recipient warm ischemia time (WIT). Additionally, we prefer to revascularize simultaneously, because sequential revascularization requires added WIT and increases the risk of troublesome bleeding (14).

Prolonged WIT has been shown to have a detrimental effect on early graft function and long-term graft survival in LDKT (16–21). A study by Khan et al showed that WIT greater than 45 min was a risk factor for poor early graft function; they also reported that longer WIT was likely attributed to performance of multiple anastomoses in MRA donors (19). Similarly, Marzouk et al reported that an anastomosis time greater than 29 min was associated with an increased need for dialysis and length of stay, as well as slower recovery of kidney function (20). Additionally, Weissenbacher et al demonstrated that an anastomosis time greater than 30 min significantly affects long-term graft outcome and leads to inferior patient survival (21). In this current study, we describe in detail 18 different techniques for reconstruction of MRA in LDKT with the goal of minimizing both WIT and the risks associated with performing these complex anastomoses. Surgical loupes at 3.5× magnification were used for the reconstructions, which have been shown to increase the ease of performing anastomosis and yield better results in living-donor transplantation (32).

Of the reconstructions where a single renal artery lumen was created (*N* = 69), we report a median WIT of 27 min. In the four cases where vessels were implanted with two arterial anastomoses, the median WIT was 31.5 min. Our median WIT for creating a single inflow orifice is acceptable compared to the reported published literature (19–21, 33). We report no incidence of DGF nor vascular or urological complications in any of our patients during the first 12 months post-transplant.

Our main goal of the study was not reached in these four cases, because the accessory polar artery was located too far from the main renal artery to be safely reconstructed into a single lumen. Therefore, the accessory polar artery was anastomosed separately to other suitable vessels located a shorter distance away from it compared with the main renal artery. The use of interposition grafting to extend the length of the polar arteries (which we implemented in seven cases of short arteries) was not an option for achieving a single lumen in these specific cases, as it would have required too long of a graft, increasing the risk of complications. Nevertheless, the use of interposition grafting in LDKT has been shown to be a useful standard method for grafts with MRA (9, 10, 34). A study by Hiramitsu et al (10) describe the usefulness of arterial reconstruction using the recipient’s own internal iliac artery for MRA grafts. They report no significant differences in complication incidence or perioperative and postoperative graft function of the interposition group at 60 months of follow-up compared to the conjoined group and the end-to-side method group. A few reports in the literature describe the use of donor gonadal vein as a conduit for renal arteries in LDKT with no vascular complications noted during short-term follow-up of these cases; however, long-term patency and safety remain unclear (9, 35–37). In our cohort, interposition grafting was performed in seven cases with various conduits such as recipient inferior epigastric artery, recipient internal iliac artery, deceased donor external iliac artery, and donor gonadal vein with no observed vascular or post-operative complications as of last follow-up.

When dealing with deceased donor kidney grafts with MRA, we also perform vascular reconstructions with the goal of creating a single arterial orifice in efforts to minimize ischemic insult. We commonly transplant MRA from deceased donors with the use of a Carrel aortic patch. If the renal arteries are located too far apart from the aorta and result in a case of long Carrel patch, we trim the Carrel patch and anastomosis it end-to-end to create a shorter carrel patch (38), or we perform a back-table vascular reconstruction into a single ostium for the same reasons as indicated in this manuscript.

Limitations of our study include the lack of comparison to outcomes for LDKT of single renal arteries. Additionally, sample sizes for certain subgroups of patients were relatively small, limiting our ability to show significant differences between the WIT of single and multiple arterial anastomoses. Another limitation of our study includes the fact that this was an evaluation of consecutively transplanted living donor recipients performed at a single center by a single, highly experienced transplant surgeon. While the chances of achieving such successful anastomoses without post-operative complications being an issue requires a surgeon who is highly experienced in performing such techniques, these techniques can be easily duplicated and incorporated by other transplant surgeons to expand their surgical armamentarium.

## Conclusion

Complex reconstruction techniques to create a single renal artery ostium for graft implantation anastomosis in allografts with MRA shows good clinical outcomes and acceptable WIT, with no increased post-operative vascular or urological complications. These techniques can be applied by other transplant surgeons when faced with vessel multiplicity to avoid potential complications associated with multiple arterial implantations.

## Data Availability

The raw data supporting the conclusion of this article will be made available by the authors, without undue reservation.
